# Editorial

**Published:** 1902-06-15

**Authors:** 


### EDITORIAL.

The New York State Dental Society at its recent meeting started a fund by private subscriptions, which already amounts to one thousand dollars, for encouraging scientific research in dental subjects. Exactly what form it will take or how it will be administered is as yet unknown. It is suggested by the editor of the *Items of Interest*, that it should be largely increased by enlisting other individuals and dental organizations, and a board of management made up of a representative from each contributing society be raised to have charge of it. Another suggestion is that an effort be made to have a dental scientist connected with the Carnegie University to be established under the United States government auspices at Washington, and that this fund shall be used in connection with that organization. There is no question but that such work is needed, and that some such method of securing it seems at the present time the only hopeful outlook for securing it. We have no doubt the profession will respond liberally to any well-digested plan for accomplishing this purpose. But we believe it would be much better if some definite plan were first adopted so that the future management may not be hampered by conflicting ideas which may detract from that persistent and consecutive effort which is essential to the accomplishment of any scientific work of real value or importance.

In connection with the idea of raising a fund to stimulate scientific investigations along dental lines, why can not something be done to have the funds belonging to the estate of the late Dr. Evans, used to encourage the object? We are not familiar with the specific terms of the will, but it would seem that this work could be made to harmonize with almost any object which might have been in the mind of one who was so greatly interested in the higher aims of his profession, at least it might be made a factor in it.

We hoped to print in this issue the new dental law for Ohio, but have not been able to get a corrected copy of the law as it finally passed the legislature. It is hoped that some of the weak places in the old law have been made good, and that it will be a much more efficient law than we have ever had before. The new board was named by the Governor June 5th, and is as follows: Dr. H. C. Brown, Columbus; Dr. J. K. Douglas, Sandusky; Dr. L. L. Barber, Toledo; Dr. Henry Barnes, Cleveland; Dr. Stanley Smith, Cincinnati. We are acquainted with all the members, except one, and they are men of high professional character, which will assure a proper administration of the law. The retiring board was one of the best the State ever had and made the best of the law as it found it.

The Illinois State Dental Society met at Springfield this year and elected the following officers for the ensuing year: A. H. Peck, President; H. J. Goslee, Secretary; Treasurer, C. N. Johnson. The society will meet next year at Bloomington, Ill.

